# Risk factors and outcome variables of cardiorenal syndrome type 1 from the nephrologist’s perspective

**DOI:** 10.1007/s11255-021-03036-w

**Published:** 2021-10-28

**Authors:** Dominik Seckinger, Oliver Ritter, Daniel Patschan

**Affiliations:** Klinik Für Kardiologie, Angiologie und Nephrologie, Klinikum Brandenburg, Medizinische Hochschule Brandenburg, Hochstraße 29, 14770 Brandenburg, Germany

**Keywords:** CRS type 1, Mortality, Dialysis, Recovery of kidney function

## Abstract

**Background and aim:**

In cardiorenal syndrome (CRS) type 1, acute cardiac failure or acute decompensation of chronic heart failure causes acute kidney injury (AKI). Every individual AKI episode increases the risk for chronic kidney disease (CKD) in the long term. In this study, we aimed to evaluate epidemiological characteristics and outcome variables of CRS type 1 individuals from the nephrologist’s perspective.

**Methods:**

The study was performed in a retrospective, observational manner. All AKI patients treated at the Brandenburg Hospital of the Medical School of Brandenburg between January and December 2019 were screened for diagnostic criteria of CRS type 1. Endpoints were in-hospital death, need for dialysis, and renal recovery.

**Results:**

During the screening, 198 out of 1189 (16.6%) AKI subjects were assigned to the diagnosis CRS type 1. The overall in-hospital mortality was 19.2%; 9.6% of the patients required dialysis due to AKI. Complete recovery of kidney function was observed in 86 individuals (43.4%); incomplete recovery occurred in 55 patients (27.8%). Mortality-predictive variables were AKIN stage 2, longer ICU treatment, and insulin-dependent diabetes. Regarding dialysis, AKIN stage 3 and higher potassium at the time of diagnosis were predictive. Subjects with longer in-hospital stay recovered more often from CRS type 1.

**Conclusions:**

The incidence of CRS type 1 is high (∼16% of all in-hospital AKI subjects) and the mortality is higher than the average mortality of AKI in general. At the same time, complete recovery of kidney function occurs less frequent. The kidney-related follow-up management of CRS type 1 needs to be significantly optimized to improve the long-term outcome of affected patients.

## Introduction

In 2008, Ronco et al. introduced the concept of cardiorenal syndromes (CRS) [1]. It differentiates between five distinct CRS types, from which all are characterized by a simultaneous affection of both heart and kidney function/structure in an either acute or chronic manner. The principal idea was (and still is) to emphasize the inter- or even multidisciplinary character of the diseases, not only from a pathophysiological but also a therapeutic perspective.

In CRS type 1, acute cardiac failure or acute decompensation of chronic heart failure impairs kidney function [2]. Impaired kidney function typically occurs as acute kidney injury (AKI). The 2012 published version of the ‘KDIGO clinical practice guidelines for acute kidney injury’ summarizes respective diagnostic criteria [3]. In general, AKI evolves in up to 30% of all hospitalized subjects in central Europe and the US [4]. It represents a major challenge for physicians all over the world, since early diagnosis is difficult and therapeutic measures are limited to say the least [5–7]. In addition, every individual AKI epidose increases the risk for chronic kidney disease (CKD) later in life [8–11].

It is being estimated that ∼25% of all subjects that are hospitalized due to acute decompensated heart failure develop acute kidney dysfunction or AKI of variable severity (CRS type 1) [12–14]. Lately, an Indian study [15] evaluated in-hospital patients treated between October 2017 and September 2019. Two-hundred and fifteen subjects were diagnosed with acute heart failure; 47 individuals (21%) also suffered from AKI which led to the diagnosis CRS type 1. The most common risk factor was coronary artery disease; 42.5% of all CRS type 1 patients did not survive. Further studies retrospectively analyzed CRS epidemiology in a large cohort of patients investigated by echocardiography [16] and in subjects treated under intensive care conditions (longitudinal design [17]). Both studies revealed CRS in general but more so CRS type 1 as mortality risk factor. Finally, a Chinese study by Hu et al. [18] retrospectively characterized CRS type 1 outcome and risk factors in older subjects (> = 60 years of age). The following parameters were associated with a lower chance of survival: use of diuretics, beta blockers, and dialysis. Other studies also emphasized the deleterious effects of certain CRS types and particularly of CRS type 1 on the overall prognosis of hospitalized patients. Nevertheless, the data on kidney-related outcome variables are still limited. Those are persistent dialysis dependency, recovery of kidney function until demission or death, and AKI-related follow-up recommendations. Regarding the substantial impact of every AKI epidose on mortality in the short term and on the risk for acquiring CKD in the long term, such informations are essential. In this study, we aimed to evaluate epidemiological characteristics and outcome variables of CRS type 1 individuals from the nephrologist’s perspective. The study was performed in a retrospective, observational manner.

## Methods

### Design

The study was conducted in a monocentric, retrospective, and observational manner. It was not required to obtain written consent due to the retrospective nature of the study. The study was formally approved by the ethics committee of the Medical School of Brandenburg (No.: E-01-20200602). The observational period lasted from January until December 2019. Based on an electronic algorithm which was implemented hospital-wide in summer 2018, every patient with an increase of serum creatinine according to criteria 1 or 2 of the KDIGO guideline from 2012 [3] was screened. Subjects were included if they met the definition criteria for CRS type 1. Additional inclusion criteria were: age >  = 18 years and in-hospital treatment for a minimum of 2 days. If AKI occurred more than once per in-hospital treatment period, only one AKI episode was considered. Not included were subjects with pre-existing CKD 5D or with CHF stage 4 according to the NYHA classification. Other exclusion criteria were any circumstances that potentially may have caused AKI apart from cardiac insufficiency: sepsis, nephrotic syndrome, fluid/blood loss, or hepatorenal syndrome.

### Definition of CRS type 1

The diagnosis of CRS type 1 was made if AKI according to the KDIGO guideline [3] occurred secondary to a cardiac event with acute onset. A cardiac event with acute onset was presumed if one or more out of three scenarios was/were present: (1) dyspnea and/or cyanosis including symptoms of congestion (peripheral edema and jugular vein distension), (2) radiographic signs of pulmonary congestion, (3) one or more of the following echocardiographic findings: diminished left-ventricular ejection fraction, regional wall motion abnormalities, and left-ventricular valve dysfunction of grade 2 or higher. The definition and thus inclusion criteria for study participants were exclusively checked and verified by one nephrologist.

### Endpoints

Primary endpoint was in-hospital survival. Secondary endpoints were the need for dialysis and recovery of kidney function until demission (either alive or dead). Complete renal recovery was diagnosed if the last eGFR was ≤ 10% the initial eGFR. If the last eGFR was higher than the lowest value during the treatment course but more than 10% lower as compared to the initial value, we diagnosed incomplete recovery.

### Statistics

Comparisons between two groups were performed with Chi-square test for categorical data. Numerical data were initially tested for normal distribution with the Kolmogorov–Smirnov test. Normally distributed data were compared with Student’s *t* test (two groups) or with ANOVA (three groups), not normally distributed data were compared with the Mann–Whitney test (two groups) or the Kruskal–Wallis test (three groups). For correlation analysis, we calculated the Spearman’s rank correlation coefficient. For the identification of endpoint risk factors, we performed multivariate logistic regression analysis. A *p *value of below 0.05 was stated as statistically significant. Results are either given in percent or as mean ± SD or SEM as indicated. For all statistical analyses, we employed the following application: Wizard 2 for MacOS, Version 2.0.5, developer: Evan Miller.

## Results

### Patients

During the screening period between January and December 2019, a total number of 1,189 subjects were diagnosed with acute kidney injury according to KDIGO [3]. One-hundred ninety-eight (198–16.6%) out of these patients were assigned to the diagnosis CRS type 1 according to the criteria defined in the Methods section. Eighty-two were females; 116 were males. The mean age of all patients was 78.2 ± 9.4 years. The mean duration of in-hospital treatment was 16.3 ± 10.5 days. The severity of AKI according to the AKIN [19] classification was: stage 1 *n* = 136 (68.7%), stage 2 *n* = 38 (19.2%), and stage 3 *n* = 24 (12.1%). All further characteristics of included subjects are summarized in Table 1.

**Table 1:** Patients’ baseline characteristics

Variable	Baseline characteristics
Gender (females/males)	82 (41.4%)/116 (58.6%)
Age (mean years ± SD)	78.2 ± 9.4
In-hospital stay (mean days ± SD)	16.3 ± 10.5
AKIN stage (1/2/3)	136 (68.7%)/38 (19.2%)/24 (12.1%)
Initial eGFR (ml/min ± SD)	43.6 ± 20
Minimal eGFR (ml/min ± SD)	24.77 ± 12.0
eGFR at demission (ml/min ± SD)	38.8 ± 21.6
Initial sodium (mMol/L ± SD)	137.7 ± 5.1
Sodium at AKI onset (mMol/L ± SD)	138.7 ± 5.2
Initial potassium (mMol/L ± SD)	4.4 ± 0.7
Potassium at AKI onset (mMol/L ± SD)	4.4 ± 0.7
Initial CRP (mg/L ± SD)	36 ± 56
Peak CRP (mg/L ± SD)	98 ± 88
NT-proBNP (pg/mL ± SD)	12,101 ± 12,169
Vasopressors	48 (24.2%)
Ventilation (no/non-invasive/invasive)	154 (77.8%)/26 (13.1%)/18 (9.1%)
ICU treatment (no/1–3/4–10/ > 10 days)	146 (73.7%)/21 (10.6%)/19 (9.6%)/12 (6.1%)
Coronary angiography (no/before/after AKI onset)	145 (73.2%)/26 (13.1%)/27 (13.6%)
Hypertension	173 (88.7%)
Diabetes (no/non-insulin-dependent/insulin-dependent)	91 (46.2%)/28 (14.2%)/78 (39.6%)
Pre-existing CKD	127 (64.5%)
Pre-existing CHF	110 (56.4%)
Pre-existing CAD	102 (51.5%)
Obesity	102 (51.5%)
BMI (mean in mg/qm ± SD)	30.3 ± 9.8
Hyperuricemia	45 (23%)
Neoplasia	50 (25.6%)

### In-hospital survival

**Fig. 1:**
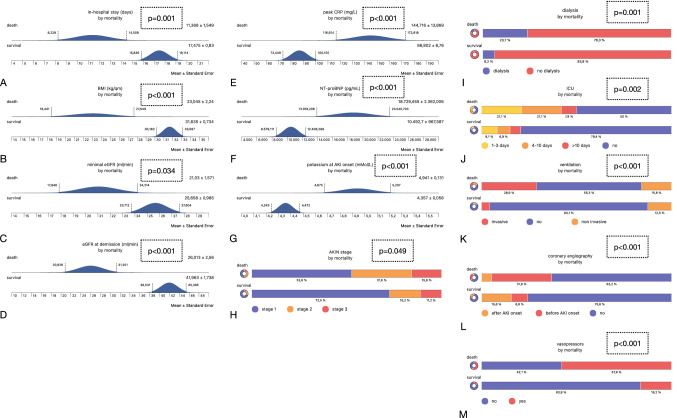
Summary of all significant findings of the mortality analyses. **A** Duration of in-hospital treatment (mean days ± SEM); **B** body mass index (kg/qm); **C** initial eGFR (ml/min); **D** eGFR at demission (ml/min); **E** peak CRP (mg/l); **F** initial NT-proBNP (pg/ml); **G** serum potassium at AKI onset (mMol/L); **H** AKIN stages (1–3); **I** dialysis (yes/no); **J** ICU treatment (no/1–3 days/4–10 days/more than 10 days); **K** ventilatory therapy (no/non-invasive/invasive); **L** coronary angiography (no/before AKI onset/after AKI onset); **M** vasopressor therapy (yes/no)

The overall in-hospital mortality was 19.2% (*n* = 38). To identify surrogate parameters that were possibly associated with a higher risk for death, the following variables were defined: gender, age, duration of in-hospital treatment, initial eGFR and eGFR at demission (either alive or dead), dialysis therapy at any time during hospital treatment, initial serum sodium and potassium, serum sodium and potassium at the time of AKI diagnosis (onset), initial and peak CRP, initial NT-proBNP, vasopressor therapy at any time during hospital treatment, ventilatory therapy at any time during hospital treatment (non-invasive and invasive), ICU treatment (categorized according to the length of stay at the ICU), coronary angiography, diabetes mellitus, pre-existing chronic kidney disease (CKD)/chronic heart failure (CHF)/coronary artery disease (CAD), obesity including the body mass index (BMI), hyperuricemia, and neoplasia (currently or in the past). The following differences were identified: the in-hospital treatment time was longer in surviving subjects, AKIN stages 2 and 3 were diagnosed more often in non-survivors, and both, the minimal eGFR and the eGFR at the time of demission were lower in non-survivors. Dialysis was performed more often in non-survivors, serum potassium at the time of AKI onset was lower in survivors, and comparable differences were found for peak CRP and NT-proBNP. Vasopressor therapy was initiated more frequently in non-survivors; the latter also required (non-invasive and invasive) ventilation more often. ICU therapy became mandatory in fewer survivors; in addition, coronary angiography was performed less frequently in surviving individuals. Finally, the average weight was higher in surviving subjects. Since the differences seen between survivors and non-survivors may not be proposed as mortality risk factors per se, additional multivariate logistic regression analysis included the following variables: age, male gender, in-hospital stay, AKIN stage 2, minimal EGFR, peak CRP, NT-proBNP, ICU treatment for longer than 10 days, insulin-dependent diabetes, the absence of CKD or CHF, BMI, and a negative history neoplasia. Three variables were identified as positive predictors of survival: in-hospital-stay, BMI, and negative history of neoplasia; three variables however were negatively predictive: AKIN stage 2, ICU treatment for more than 10 days, and insulin-dependent diabetes. Tables 2 and 3 and Fig. 1 summarize all results of the mortality analyses. Table 3 also lists odds ratios, the 95% confidence intervals, and *p *values of the multivariate analysis.

**Table 2:** Mortality

Variable	Survival	Death	*p* value
Gender (females in %)	41.2	42.1	0.92
Age (mean years)	77.9 ± 0.7	79.7 ± 1.8	0.28
In-hospital stay (mean days)	17.4 ± 0.8	11.3 ± 1.5	**0.001**
AKIN stage (1/2/3 in %)	72.5/16.2/11.2	52.6/31.6/15.8	**0.049**
Initial eGFR (ml/min)	43.9 ± 1.6	42.4 ± 2.7	0.67
Minimal eGFR (ml/min)	25.6 ± 0.9	21 ± 1.5	**0.034**
eGFR at demission (ml/min)	41.9 ± 1.7	26 ± 2.5	**< 0.001**
Dialysis (%)	6.2	23.7	**0.001**
Initial sodium (mMol/L)	137.7 ± 0.4	137.8 ± 0.8	0.9
Sodium at AKI onset (mMol/L)	138.5 ± 0.4	139.6 ± 1.1	0.26
Initial potassium (mMol/L)	4.4 ± 0.05	4.4 ± 0.1	0.9
Potassium at AKI onset (mMol/L)	4.3 ± 0.05	4.9 ± 0.13	**< 0.001**
Initial CRP (mg/L)	32.2 ± 3.9	52.03 ± 12.3	0.05
Peak CRP (mg/L)	86.8 ± 6.7	144.7 ± 13.8	**< 0.001**
NT-proBNP (pg/mL)	10,492 ± 967	18,729 ± 2362	**< 0.001**
Vasopressors (%)	16.2	57.9	**< 0.001**
Ventilation (no/non-invasive/invasive in %)	83.1/12.5/4.4	55.3/15.8/28.9	**< 0.001**
ICU treatment (no/1–3/4–10/ > 10 days in %)	79.4/8.1/6.9/5.6	50/21.1/21.1/7.9	**0.002**
Coronary angiography (no/before/after AKI onset in %)	75.6/8.8/15.6	63.2/31.6/5.3	**< 0.001**
Hypertension (%)	88.6	89.2	0.9
Diabetes (no/non-insulin-dependent/insulin-dependent in %)	46.2/14.4/39.4	45.9/13.5/40.5	0.98
Pre-existing CKD (%)	66.2	56.8	0.27
Pre-existing CHF (%)	54.7	63.9	0.31
Pre-existing CAD (%)	50.6	55.3	0.6
Obesity (%)	53.5	44.7	0.33
BMI (mean in mg/qm)	31.6 ± 0.7	23 ± 2.2	**< 0.001**
Hyperuricemia (%)	24.5	16.2	0.27
Neoplasia (%)	24.5	30.6	0.45

Twenty-eight variables were compared between survivors and non-survivors. Results are either shown as mean ± SEM or as percentages

Statistically significant *p* values are in bold (*p* < 0.05)

**Table 3:** Multivariate logistic regression analysis regarding mortality

Variable	Odds ratio	95% Confidence interval	*p* value
Age	0.968	(0.928, 1.01)	0.132
Male gender	2.783	(0.76, 10.19)	0.122
In-hospital stay	1.098	(1.005, 1.201)	**0.039**
AKIN stage 2	0.19	(0.046, 0.787)	**0.022**
Minimal eGFR (mL/min)	1.015	(0.936, 1.1)	0.721
Peak CRP (mg/L)	0.994	(0.987, 1.001)	0.094
NT-proBNP (pg/mL)	1–1.456e-5	(1–7.137e-5, 1 + 4.224e-5)	0.615
ICU treatment (> 10 days)	0.013	(0, 0.35)	**0.01**
Diabetes (insulin-dependent)	0.179	(0.039, 0.83)	**0.028**
No pre-existing CKD	1.088	(0.285, 4.15)	0.902
No pre-existing CHF	1.621	(0.478, 5.492)	0.438
Bmi (mg/qm)	1.137	(1.023, 1.264)	**0.017**
No neoplasia	5.105	(1.221, 21,349)	**0.026**

Three variables were identified as positive predictors of survival: in-hospital-stay, BMI, and negative history of neoplasia; three variables however were negatively predictive: AKIN stage 2, ICU treatment for more than 10 days, and insulin-dependent diabetes

Statistically significant *p* values are in bold (*p* < 0.05)

### Dialysis

**Fig. 2:**
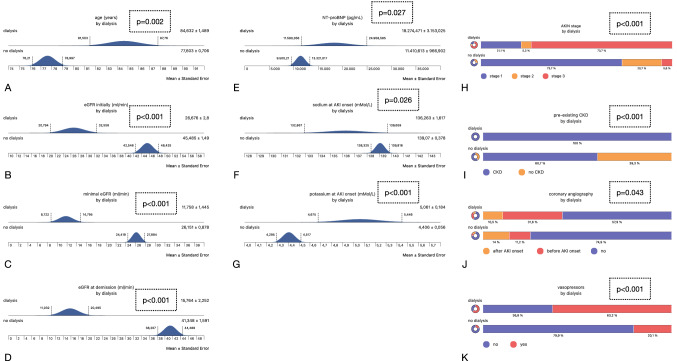
Summary of all significant findings of the dialysis-related analyses. **A** Age (years ± SEM). **B** initial eGFR (ml/min); **C** minimal eGFR (ml/min); **D** eGFR at demission (ml/min); **E** initial NT-proBNP (pg/ml); **F** serum sodium at AKI onset (mMol/L); **G** serum potassium at AKI onset (mMol/L); **H** Akin stage (1–3); **I** pre-existing CKD (yes/no); **J** coronary angiography (no/before AKI onset/after AKI onset); vasopressor therapy (yes/no)

A total number of 19 (9.6%) patients required dialysis therapy due to CRS type 1-associated AKI. Forty-seven (47.4) % of the patients did not survive as opposed to 16.2% of individuals without dialysis (*p* = 0.001). For risk analysis, we employed the same variables as in the previous section. Table 3 and Fig. 2 summarize all findings. The following differences between subjects without (ND–no dialysis) and with dialysis therapy (D–dialysis) were identified: ND subjects were younger and were more often diagnosed with lower AKI stages according to AKIN. ND patients also showed a higher eGFR initially, at the minimum and at the time of demission. Serum potassium was lower in ND patients at the time of AKI onset. The same difference was identified for NT-proBNP and for vasopressor therapy. Patients without the need for dialysis underwent coronary angiography less frequent. Pre-existing CKD was diagnosed in all D patients as opposed to 60.7% of non-dialyzed subjects. Additional multivariate logistic regression analysis included the following variables: age, male gender, in-hospital stay, AKIN stage 3, peak CRP, NT-proBNP, and both, serum sodium and potassium initially and at the time of AKI onset. AKIN stage 3 and potassium at AKI onset were identified as predictive for dialysis. Tables 4 and 5 and Fig. 2 summarize all findings.

**Table 4:** Dialysis

Variable	No dialysis	Dialysis	*p* value
Gender (females in %)	40.8	47.4	0.57
Age (mean years)	77.6 ± 0.7	84.6 ± 1.4	**0.002**
In-hospital stay (mean days)	16 ± 0.7	19 ± 2.8	0.32
AKIN stage (1/2/3 in %)	73.7/20.7/5.6	21.1/5.3/73.7	**< 0.001**
Initial eGFR (ml/min)	45.4 ± 1.5	26.6 ± 2.8	**< 0.001**
Minimal eGFR (ml/min)	26.1 ± 0.8	11.7 ± 1.4	**< 0.001**
eGFR at demission (ml/min)	41.3 ± 1.6	15.7 ± 2.2	**< 0.001**
Initial sodium (mMol/L)	137.8 ± 0.3	136.7 ± 1.4	0.46
Sodium at AKI onset (mMol/L)	139 ± 0.37	136.2 ± 1.6	0.087
Initial potassium (mMol/L)	4.4 ± 0.05	4.6 ± 0.17	0.6
Potassium at AKI onset (mMol/L)	4.4 ± 0.05	5 ± 0.18	**< 0.001**
Initial CRP (mg/L)	34.4 ± 3.9	51.1 ± 18.7	0.52
Peak CRP (mg/L)	96.2 ± 6.5	114.7 ± 21.6	0.28
NT-proBNP (pg/mL)	11,410 ± 966	18,274 ± 3,153	**0.016**
Vasopressors (%)	20.1	63.2	**< 0.001**
Ventilation (no/non-invasive/invasive in %)	78.2/12.8/8.9	73.7/15.8/10.5	0.9
ICU treatment (no/1–3/4–10/ > 10 days in %)	74.3/11.2/8.4/6.1	68.4/5.3/21.1/5.3	0.31
Coronary angiography (no/before/after AKI onset in %)	74.9/11.2/14	57.9/31.6/10.5	**0.04**
Hypertension (%)	89.2	84.2	0.5
Diabetes (no/non-insulin-dependent/insulin-dependent in %)	45.5/14.6/39.9	52.6/10.5/36.8	0.8
Pre-existing CKD (%)	60.7	100	**< 0.001**
Pre-existing CHF (%)	55.7	63.2	0.53
Pre-existing CAD (%)	50.8	57.9	0.55
Obesity (%)	51.7	52.6	0.93
BMI (mean in mg/qm)	30.6 ± 0.7	27 ± 3.2	0.73
Hyperuricemia (%)	23.2	21.2	0.83
Neoplasia (%)	26.1	21.1	0.63

As opposed to the mortality analyses, 27 variables were analyzed (dialysis excluded). Results are either shown as mean ± SEM or as percentages

Statistically significant *p* values are in bold (*p* < 0.05)

**Table 5:** Multivariate logistic regression analysis regarding dialysis

Variable	Odds ratio	95% Confidence interval	*p* value
Age	0.918	(0.831, 1.015)	0.096
Male gender	1.666	(0.453, 6.13)	0.443
In-hospital stay	0.973	(0.972, 1.021)	0.262
AKIN stage 3	0.026	(0.008, 0.101)	**< 0.001**
Peak CRP (mg/L)	− 1.219E-4	(− 0.007, 0.007)	0.972
NT-proBNP (pg/mL)	− 3.45e-5	(− 7.72e-5, 8.207e-6)	0.113
Initial sodium (mMol/L)	− 0.075	(− 0.224, 0.073)	0.322
Sodium at AKI onset (mMol/L)	0.151	(− 0.028, 0.33)	0.098
Initial potassium (mMol/L)	0.01	(− 0.863, 0.883)	0.982
Potassium at AKI onset (mMol/L)	− 1.015	(− 1.864, − 0.166)	**0.019**

AKIN stage 3 and potassium at AKI onset were identified as predictive

Statistically significant *p* values are in bold (*p* < 0.05)

### Recovery of kidney function

**Fig. 3:**
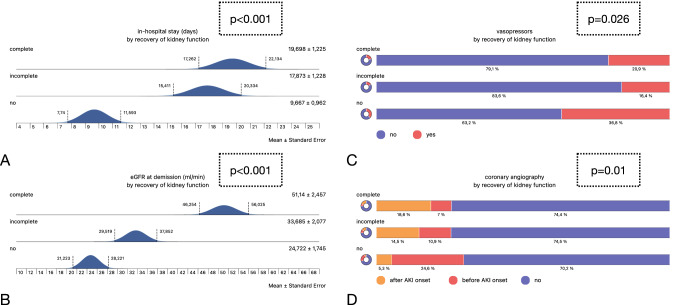
Summary of all significant findings of the recovery analyses. **A** Duration of in-hospital treatment (mean days ± SEM); **B** eGFR at demission (ml/min); **C** vasopressor therapy (yes/no); **D** coronary angiography (no/before AKI onset/after AKI onset)

According to the criteria defined in the Methods section, complete recovery of kidney function (CR) occurred in 86 individuals (43.4%), incomplete recovery (IR) was diagnosed in 55 patients (27.8%). Fifty-seven patients (28.8%) did not recover at all (NR). Patients with versus without recovery differed in the following variables: the in-hospital treatment time was longer in CR/IR as opposed to NR patients. Vasopressor therapy was performed less frequent in patients with complete or incomplete recovery. Finally, coronary angiography was performed more often in subjects without recovery. Multivariate logistic regression analysis included the same variables as mentioned under the ‘Dialysis’ section. Two variables were predictive: in-hospital stay (positive) and AKIN stage 3 (negative) (Tables 6 and 7 and Fig. 3).

**Table 6:** Recovery of kidney function

Variable	Recovery (complete/incomplete)	No recovery	*p* value
Gender (females in %)	37.2/45.5	37.2	0.56
Age (mean years)	77.6 ± 0.9/79.2 ± 1.1	78.2 ± 1.4	0.55
In-hospital stay (mean days)	19.6 ± 1.2/17.8 ± 1.2	9.6 ± 0.9	**< 0.001**
AKIN stage (1/2/3 in %)	76.7/18.6/4.7 and 63.6/20/16.4	61.4/19.3/19.3	0.07
Initial eGFR (ml/min)	41.3 ± 2.2/47.1 ± 2.7	43.8 ± 2.4	0.2
Minimal eGFR (ml/min)	25.6 ± 1.3/24.4 ± 1.5	23.8 ± 1.6	0.55
eGFR at demission (ml/min)	51.1 ± 2.4/33.6 ± 2	24.7 ± 1.7	**< 0.001**
Dialysis (%)	4.7/10.9	15.8	0.08
Initial sodium (mMol/L)	137.3 ± 0.56/138 ± 0.76	138 ± 0.64	0.26
Sodium at AKI onset (mMol/L)	138.6 ± 0.6/138.6 ± 0.6	139.2 ± 0.72	0.41
Initial potassium (mMol/L)	4.4 ± 0.08/4.3 ± 0.08	4.5 ± 0.09	0.18
Potassium at AKI onset (mMol/L)	4.4 ± 0.08/4.3 ± 0.09	4.6 ± 0.1	0.056
Initial CRP (mg/L)	36 ± 6.1/35.7 ± 6.6	36.4 ± 8.3	0.59
Peak CRP (mg/L)	106.7 ± 10.1/82.2 ± 9.5	100.1 ± 12.4	0.47
NT-proBNP (pg/mL)	11,867 ± 1,368/10,514 ± 1,678	14,328 ± 1,961	0.23
Vasopressors (%)	20.9/16.4	36.8	**0.026**
Ventilation (no/non-invasive/invasive in %)	76.7/16.3/7 and 85.5/10.9/3.6	71.9/10.5/17.5	0.07
ICU treatment (no/1–3/4–10/ > 10 days in %)	70.9/7/12.8/9.3 and 83.6/9.1/3.6/3.6	68.4/17.5/10.5/3.5	0.1
Coronary angiography (no/before/after AKI onset in %)	74.4/7/18.6 and 74.5/10.9/14.5	70.2/24.6/5.3	**0.01**
Hypertension (%)	90.6/88.9	85.7	0.6
Diabetes (no/non-insulin-dependent/insulin-dependent in %)	45.3/12.8/41.9 and 47.3/18.2/34.5	46.4/12.5/41.1	0.8
Pre-existing CKD (%)	65.1/63.6	64.3	0.9
Pre-existing CHF (%)	57.1/54.5	57.1	0.9
Pre-existing CAD (%)	54.7/50.9	47.4	0.69
Obesity (%)	48.2/40	56.1	0.23
BMI (mean in mg/qm)	29.7 ± 0.8/32.3 ± 1	28.9 ± 2	0.1
Hyperuricemia (%)	23.5/20	25	0.81
Neoplasia (%)	23.8/23.6	30.4	0.63

Results are either shown as mean ± SEM or as percentages

Statistically significant *p* values are in bold (*p* < 0.05)

**Table 7:** Multivariate logistic regression analysis regarding recovery of kidney function

Variable	Odds ratio	95% Confidence interval	*p* value
Age	0.013	(− 0.027, 0.053)	0.518
Male gender	0.213	(− 0.529, 0.956)	0.547
In-hospital stay	0.194	(0.125, 0.263)	**< 0.001**
AKIN stage 3	− 1.725	(− 2.885, − 0.566)	**0.004**
Peak CRP (mg/L)	− 0.001	(− 0.006, 0.003)	0.547
NT-proBNP (pg/mL)	− 1.113e-5	(− 4.228e-5, 2.002e-5)	0.484
Initial sodium (mMol/L)	− 0.021	(− 0.119, 0.078)	0.684
Sodium at AKI onset (mMol/L)	− 0.078	(− 0.18, 0.023)	0.131
Initial potassium (mMol/L)	− 0.118	(− 0.696, 0.46)	0.689
Potassium at AKI onset (mMol/L)	− 0.455	(− 1.033, 32.121)	0.124

In-hospital stay (positive) and AKIN stage 3 (negative) were predictive

Statistically significant *p* values are in bold (*p* < 0.05)

### Documentation of the diagnosis

Sixty-four (32.2%) demission letters did not contain any cardiorenal diagnosis at all. ‘AKI’ alone was documented in 3 letters (1.5%), ‘AKI + HF’ (HF–heart failure) was named 60 times (30.3%), ‘CKD + HF’ was documented in 57 (28.8%) letters, and the exclusive diagnosis of ‘CKD’ was documented 8 times (4%). Finally, only 6 distinctive letters (3%) contained the diagnosis ‘CRS’.

### Follow-up recommendations

Follow-up recommendations were given in only 14 patients (8%).

## Discussion

The current study reports on epidemiology and outcome of CRS type 1 patients treated at a cardiorenal unit over the year 2019. We particularly intended to evaluate outcome variable from the nephrologist’s perspective. One-hundred ninety-eight (198) out of 1189 AKI patients were diagnosed with CRS 1 (16.2%).

Mortality. The primary endpoint (in-hospital mortality) was reached in 19.2% (*n* = 38). Mortality data in CRS type 1 are available from both, retrospective and prospective trials. In a 2012 published, retrospective study, AKI was diagnosed according to the RIFLE criteria [20, 21]. The in-hospital mortality was 16.7% without pre-existing CKD and 24.5%, if kidney function was already reduced at the time of admission. Shirakabe reported mortality rates of 13.8% in acute heart failure (AHF) patients with the so-called early as opposed to 11.8% of the subjects with late AKI [22]. Li et al. finally found an average mortality of 23.5% in AHF patients with AKI as compared to 7.2% of AHF subjects without deteriorated kidney function (threefold increase) [23]. These percentages were identified if the KDIGO criteria were applied for diagnosis. Earlier retrospective studies were published before 2012 when the latest version of the KDIGO guideline was released [3]. In a prospective trial, Roy et al. compared the predictive power of KDIGO, RIFLE, and AKIN criteria AHF patients [24]. The 30 day mortality was 7.3% if AKI was diagnosed according to KDIGO. Vandenberghe et al. [25] extensively reviewed epidemiological data on CRS type 1 and finally included 64 studies. The authors differentiated three mortality ranges (within 28 days, at 1 year after the diagnosis, and later than 5 years after onset). The relative risk of death decreased over time. However, it was more than fivefold increased within the first 28 days. Overall, the mortality found in our study was in line with reports from the literature. We identified a different distribution of certain risk factors between survivors and non-survivors (see Results section). Surprisingly, pre-existing CKD was not associated with lower survival, respectively. Particularly, the comparable mortalities in subjects with versus without CKD were unexpected, since chronic kidney disease is known to substantially increase the cardiovascular and overall mortality per se [26]. The study by Li et al. reported higher average mortality in CRS type 1 with pre-existing CKD [23]. All differences identified were however only observational in nature. We therefore performed additional multivariate logistic regression analysis which showed longer in-hospital-stay, higher BMI, and negative history of neoplasia as predictive for survival. AKIN stage 2, ICU treatment for more than 10 days, and insulin-dependent diabetes were predictive for non-survival. Regarding obesity, heterogeneous data on AKI outcomes in obese subjects have been published [27, 28]; reliable prospective data are missing yet. Longer ICU treatment periods in subjects with as compared to those without AKI have been reported earlier [5]; specific data on mortality of CRS type 1 subjects in relation to the length of intensive care therapy however have not been reported in the literature so far.

Dialysis. Almost 10% of all CRS type 1 patients required dialysis therapy. In addition, non-surviving subjects received renal replacement therapy (RRT) significantly more often. Only a few articles on RRT in CRS type 1 have been published. Most studies related to RRT in CRS evaluated peritoneal dialysis instead of hemodialysis/hemodiafiltration. In a 2017 published prospective trial, Ponce et al. [29] included a total number of 64 CRS type 1 subjects without defining a control group. The in-hospital mortality was 32.8% (47.4% in the current investigation). Non-survivors were older, suffered from ACS more often, and showed a more positive fluid balance after the second PD treatment session, respectively. Al-Hwiesh et al. [30] published a prospective trial in CRS type 1 with a total number of 88 patients included. One half was assigned to receive either ultrafiltration treatment or tidal PD. The primary endpoint was a composition of serum creatinine and left-ventricular ejection fraction improvement. The study showed that ultrafiltration therapy was inferior to tidal PD. Several other studies on PD and refractory heart failure ± impaired kidney function have been published, but a detailed discussion is not intended. Multivariate regression analysis showed AKIN stage 3 and higher potassium at AKI onset as predictive for dialysis. These findings were plausible without doubt.

Recovery of kidney function. Only a few data are available on CRS type 1 and recovery of kidney function until demission. This particular aspect is highly important. AKI in general has been identified as risk factor for CKD, which on the other hand dramatically worsens the long-term prognosis of respective patients. Since the landmark study be Go et al. published in 2004 [26], CKD has increasingly been recognized as one of the most potent if not the most powerful risk factor for cardiovascular morbidity and mortality. In our study, 28.8% of the patients did not recover at all, while 43.4% recovered completely and 27.8% incompletely. In the earlier cited study by Zhou et al. [21], complete or full recovery of kidney function was observed in 72.3% of patients without pre-existing CKD and in 30.7% of the subjects with CKD. Complete (full) recovery was defined as a fall of the serum creatinine concentration to or below the initial value. To reliably assess the kidney-related prognosis of CRS type 1 subjects, informations about renal recovery post-AKI in general are needed. In 2016, Kellum et al. [31] published a retrospective study which included almost 17,000 AKI patients. They identified early and complete reversal of kidney dysfunction in ∼26% of all cases. No recovery at all was observed in the same percentage of subjects (26.5%). Further patterns were recovery later than 7 days after onset, early recovery followed by relapses, and finally, relapses without recovery. In 2017, the consensus document of the ‘Acute Disease Quality Initiative (ADQI) 16 Workgroup’, titled ‘Acute kidney disease and renal recovery’ introduced the concept of AKI–AKD–CKD (AKI—days 2–7 after onset; AKD—Acute Kidney Disease—days 7–90 after onset; CKD—persistent kidney dysfunction at day 90 and later) [32]. Regarding the data by Kellum et al. [31], the total percentage of subjects with complete recovery was ∼59 (early recovery + late recovery + relapses followed by recovery) as opposed to 43.4% in our investigation. Therefore, the renal prognosis of CRS type 1 subjects is at least not superior to the renal prognosis of AKI in general. For multivariate regression analysis, we summarized subjects with complete and incomplete recovery of kidney function into one group, as compared to those without recovery (group 2). Longer in-hospital stay was positive; an AKIN stage 3 was negatively predictive for renal recovery.

The final aspect to be discussed is related to the follow-up management. Both, the final documentation of any type of cardiorenal diagnosis in demission letters and more so, respective follow-up recommendations for the monitoring of kidney function were performed/given inadequately (no diagnosis at all in 32.2%, no recommendation in 92%). In 2020, Ransley et al. [33] reported that only 6% of post-AKI patients treated at the ICU received nephrology follow-up at 3 months (9% at year 1). Therefore, the physicians’ awareness to the long-term impact of AKI on the overall morbidity is most likely inadequate in general. There is urgent need for further education in this important field of medicine, not only regarding CRS but also other types of AKI.

## Data Availability

All data are available upon request to d.patschan@gmail.com.
